# HPLC-Profiles of Tocopherols, Sugars, and Organic Acids in Three Medicinal Plants Consumed as Infusions

**DOI:** 10.1155/2014/241481

**Published:** 2014-10-07

**Authors:** Custódio Lobo Roriz, Lillian Barros, Ana Maria Carvalho, Isabel C. F. R. Ferreira

**Affiliations:** Mountain Research Centre (CIMO), ESA, Polytechnic Institute of Bragança, Campus de Santa Apolónia, Apartado 1172, 5301-855 Bragança, Portugal

## Abstract

*Pterospartum tridentatum* (L.) Willk, *Gomphrena globosa* L., and *Cymbopogon citratus* (DC.) Stapf are medicinal plants that require a more detailed chemical characterization, given the importance of their consumption as infusions. Therefore, the individual profiles in tocopherols, free sugars, and organic acids were obtained by high performance liquid chromatography (HPLC) coupled to different detectors (fluorescence, refraction index, and photodiode array, resp.). *C. citratus* revealed the highest content of *α*-, and total tocopherols, glucose, sucrose, succinic, and ascorbic acids. *P. tridentatum* presented the highest fructose and total sugars content. Otherwise, *G. globosa* showed the highest organic acids concentration. As far as we know, this is the first study reporting the mentioned chemical compounds in *G. globosa* and *C. citratus*.

## 1. Introduction

For a long time, plants represented one of the most important therapies for different diseases. Nowadays, the popular use of plants as a way of treatment is still very important for human beings [1].* Pterospartum tridentatum* (L.) Willk,* Gomphrena globosa *L., and* Cymbopogon citratus* (DC.) Stapf are examples of those plants that are widely used in folk medicine, mostly as infusions.

*P. tridentatum* (family: Fabaceae) is a European endemic species and its flowers infusion is used against liver, bladder, kidney, and rheumatism problems; it is also used for high blood pressure, cough, kidney stones, diabetes, and bronchitis [1, 2].

*G. globosa* (family: Amaranthaceae) is native from Panama and Guatemala and its aqueous extract of its purple inflorescences is good to treat bronchial asthma, acute and chronic bronchitis, and whooping cough; the infusion of the flowers is used to treat oliguria and indigestion, also as expectorant and pertussis [3, 4].

*C. citratus* (family: Poaceae) is native from the Southwest Asia and its aqueous extract (i.e., in the form of infusion) is used for the treatment of several inflammation-based pathologies, in digestive disorders, diabetes, nervous disorders, and fever [5–7]. According to Novais et al. [1], the infusion is used as gastric analgesic, intestinal anti-inflammatory, and renal antispasmodic and for gall-bladder ailments, sea-sickness, and bladder ailments.

There are several reports about the biological activity of the mentioned plants, especially concerning antioxidant activity [5, 8], that have been related to their phenolic composition [3, 9], but studies regarding the composition on primary metabolites and nutrients are scarce. Moreover, many of the commercially available samples are wild and require chemical characterization. Besides secondary metabolites/nonnutrients such as phenolic compounds, medicinal plants contain primary metabolites and nutrients (e.g., as sugars, organic acids, and tocopherols) that need to be profiled and quantified.

Mono- and oligosaccharides, with low molecular weight, and their derivatives such as sugar alcohols display a major role in the structure and function of all living cells [10]. Organic acids are involved in several biochemical pathways, including energy production and formation of precursors for amino-acid biosynthesis [11]. Vitamin E (including tocopherols) is known to be an essential micronutrient for maintaining the health and wellbeing of humans and other animals. Several studies suggest that the use of vitamin E may contribute to help lowering the risks of specific chronic and degenerative diseases such as Alzheimer's disease, some types of cancer, cataracts, and ischemic heart disease [12]. All these molecules can be determined by high performance liquid chromatography (HPLC) coupled to different detectors according to their chemical properties, namely, fluorescence for tocopherols, refraction for sugars, and UV absorption for organic acids.

Therefore, the objective of the present paper was to characterize tocopherols, sugars, and organic acids in three medicinal plants (*P. tridentatum*,* G. globosa*, and* C. citratus*), widely consumed as infusions.

## 2. Material and Methods

### 2.1. Samples

Plant material of* Pterospartum tridentatum* (L.) Willk,* Gomphrena globosa* L., and* Cymbopogon citratus* (DC.) Stapf was purchased from Ervital, a Portuguese company in Castro Daire (Portugal). This company, settled in a high diverse mountain region (Montemuro, the Natura 2000 site), markets several certified plant materials with different origin, such as sustainable wild harvesting of spontaneous local species and organic farming of exogenous species.* Pterospartum tridentatum* flowers were wildly gathered in spring 2012 (respecting plant phenology and abundance) and the other studied species were grown, also in 2012, with organic farming methods. Harvested plants were processed using in-storage and low temperature drying methods (solar heated air, average daily temperature around 30–32°C in shade conditions, and controlled relative humidity). Samples for analysis were prepared from dried plant materials provided by the company, and botanical identification was confirmed by Ana Maria Carvalho, responsible for the medicinal plant collection of the Herbarium of the Escola Superior Agrária (BRESA), of the Polytechnic Institute of Bragança (Trás-os-Montes, Portugal).

### 2.2. Standards and Reagents

HPLC-grade acetonitrile, ethyl acetate, and *n*-hexane were purchased from Fisher Scientific (Lisbon, Portugal). L-ascorbic acid, tocopherol, sugar, and organic acid standards were purchased from Sigma (St. Louis, MO, USA). Racemic tocol (50 mg/mL) was purchased from Matreya (Pleasant Gap, PA, USA). Water was treated in Milli-Q water purification system (TGI Pure Water Systems, Greenville, SC, USA).

### 2.3. Tocopherols Composition

Tocopherols were determined following a previously described procedure [13]. The equipment consisted of an integrated system with a pump (Knauer, Smartline system 1000, Berlin, Germany), degasser system (Smartline manager 5000), autosampler (Jasco AS-2057, Easton, MD, USA), and a fluorescence detector (Jasco FP-2020) programmed for excitation at 290 nm and emission at 330 nm. The chromatographic separation was achieved with a Polyamide II (250 mm × 4.6 mm i.d.) normal-phase column from YMC Waters (Dinslaken, Germany) operating at 30°C (7971 R Grace oven). The mobile phase used was a mixture of *n*-hexane and ethyl acetate (70 : 30, v/v) at a flow rate of 1 mL/min, and the injection volume was 20 *μ*L. The compounds were identified by chromatographic comparisons with authentic standards. Quantification was based on calibration curves obtained from commercial standards of each compound using the internal standard (IS) methodology; racemic tocol was used as IS. The results were expressed in *μ*g per g of dry weight.

### 2.4. Sugars Composition

Free sugars were determined by high performance liquid chromatography coupled to a refraction index detector (HPLC-RI), after an extraction procedure previously described [14]. Analysis was performed by HPLC (equipment described above) using an RI detector (Knauer Smartline 2300, Berlin, Germany). Data were analyzed using Clarity 2.4 Software (DataApex). The chromatographic separation was achieved with a Eurospher 100-5 NH2 column (4.6 × 250 mm, 5 mm, Knauer, Berlin, Germany) operating at 30°C. The mobile phase was acetonitrile/deionized water, 70 : 30 (v/v) at a flow rate of 1 mL/min. The compounds were identified by chromatographic comparisons with authentic standards. Quantification was performed using the internal standard method; melezitose was used as IS. The results were expressed in mg per g of dry weight.

### 2.5. Organic Acids

Organic acids were determined following a procedure previously described [15]. The analysis was performed using a Shimadzu 20A series UFLC (Shimadzu Corporation, Kyoto, Japan). Separation was achieved on a SphereClone (Phenomenex, Torrance, CA, USA) reverse phase C_18_ column (5 *μ*m, 250 mm × 4.6 mm i.d.) thermostated at 35°C. The elution was performed with sulfuric acid (3.6 mM) using a flow rate of 0.8 mL/min. Detection was carried out in a PDA (photodiode array detector), using 215 and 245 nm (for ascorbic acid) as preferred wavelengths. The organic acids found were quantified by comparison of the area of their peaks recorded at 215 nm with calibration curves obtained from commercial standards of each compound. The results were expressed in mg per g of dry weight.

### 2.6. Statistical Analysis

All the assays were carried out in triplicate, and the results are expressed as mean values and standard deviation (SD). The results were analyzed using one-way analysis of variance (ANOVA) followed by Tukey's HSD Test with *α* = 0.05. This treatment was carried out using SPSS v.22.0 program.

## 3. Results and Discussion

**Figure 1:**
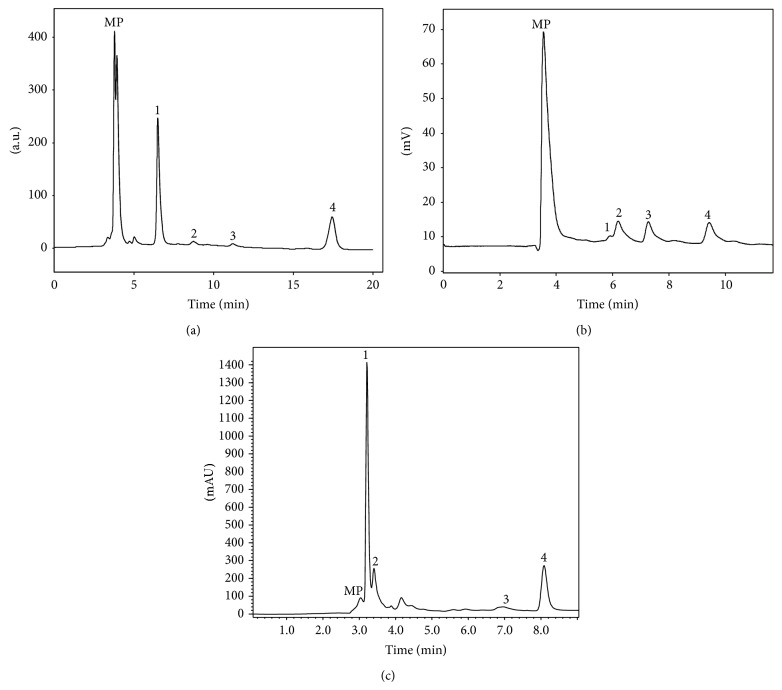
Chromatographic profile of (a) tocopherols in* Gomphrena globosa* obtained using HPLC-FD: 1: *α*-tocopherol, 2: BHT, 3: *γ*-tocopherol, and 4: tocol (IS); (b) sugars in* Cymbopogon citratus* obtained using HPLC-RI: 1: fructose, 2: glucose, 3: sucrose, and 4: melezitose (IS); (c) organic acids in* Gomphrena globosa* obtained using UFLC-PDA: 1: oxalic acid, 2: malic acid, 3: citric acid, and 4: fumaric acid. MP: mobile phase.

The chemical composition of the three plant species in tocopherols, free sugars, and organic acids is presented in Table 1.* C. citratus* gave the highest *α*- and total tocopherols content but did not present *δ*-tocopherol that was found in the other two species (e.g., Figure 1(a)). Tocopherols are lipid-soluble antioxidants, being *α*-tocopherol the most active isoform, due to its role in lipid peroxidation inhibition [16]. These molecules are widely used as functional ingredients in food, pharmaceutical, and cosmetic preparations [17]. As far as we know this is the first report on tocopherols composition of* C. citratus* and* G. globosa*; otherwise, the values obtained for* P. tridentatum* were similar to the ones described by the authors for a wild traditionally shade-dried sample (8.8 *μ*g/g dw) [8]. Furthermore, tocopherols have also been reported in other species of the Fabaceae family, such as* Cicer arietinum*,* Lathyrus sativus* [18],* Cytisus multiflorus*,* Cytisus scoparius*, and* Cytisus striatus* [8]; and the amounts found ranged between 6.3 and 23.1 mg/100 g of dw, which is in the range of the sample studied herein, detecting all the isoforms. All isoforms of tocopherols have also been reported in Poaceae and Amaranthaceae families [19], but the quantities cannot be compared due to the units in which they are expressed.

Regarding free sugars,* P. tridentatum* showed the highest levels of fructose and total sugars; nevertheless, the values obtained were much higher than the concentrations found in a wild sample previously studied (0.3 and 49.6 g/100 g dw for fructose and total sugars, resp.) [8]. This could be due to different growth conditions of the plants (e.g., variability of weather and soil characteristics are major factors affecting plant development) and to different drying processes applied, which influence moisture content and plant material quality. Pinela et al. [8] simulated consumers' traditional conditions of use (shade-drying, plant material being stored in a dark, dry place and at room temperature for 30 days); dried plant material used for analysis in this study was processed in five days under the best conditions of shade, daily temperature, and relative humidity as well as airflow rate. Furthermore, the presence of sugars has also been reported in the Poaceae family, namely, sucrose, glucose, fructose, trehalose, and raffinose, but the amounts obtained cannot be compared to the ones studied herein [20]. Some species belonging to the Fabaceae family (mentioned above) [8, 18] have also shown the presence of sugars, mainly fructose, glucose, sucrose, and trehalose, although some species revealed the presence of other sugars. The amounts found ranged between 2.56 and 18.67 g/100 g of dw; these results are much lower than the one present in this study.

It should be highlighted that fructose can display antioxidant properties due to its reducing capacity. Furthermore, sugars are one of the molecules present in plant infusions that contribute to their energetic value [21].* C. citratus* gave the highest levels of glucose and sucrose (Table 1 and Figure 1(b)). No reports were found considering sugars composition in the mentioned species or in* G. globosa*.

Concerning organic acids,* G. globosa* was the sample with the highest concentration of these compounds, mainly malic and oxalic acids (Table 1 and Figure 1(c)). Citric and succinic acids were found in higher levels in* P. tridentatum* and* C. citratus*, respectively. The latter also presented ascorbic acid, a powerful antioxidant phytochemical [22, 23]. Besides their important role in the human metabolism, organic acids have other applications; for example, citric acid is a crystal thickener in bones, succinic acid is known to help in diabetes treatment, and malic acid is reported to have a bactericidal effect [24].

Among the three analyzed species, only* P. tridentatum* was previously studied regarding organic acids composition [15]; despite the fact that similar total amount was found (8.1 mg/g dw), the profile then described was slightly different, reporting also the presence of quinic, succinic, and fumaric acids. As mentioned before, differences may be caused by distinct ecological conditions for plant development (plant material provenance is quite different) and also by different characteristics of the plant material used for analysis, as a consequence of the drying processes applied to each material. A five-day controlled in-storage process may produce a better quality plant material, in terms of color (visually confirmed), texture, and moisture content, than the traditional shade-drying techniques. All the organic acids, with the exception of quinic acid, have been previously reported in some species (mentioned above) belonging to the Fabaceae family [8, 18], although the quantities present have a large variation depending on the species.

## 4. Conclusion

Overall,* C. citratus* possessed the highest content of *α*- and total tocopherols, glucose, sucrose, succinic, and ascorbic acids.* Pterospartum tridentatum* presented the highest fructose and total sugars content. Otherwise,* G. globosa* showed the highest organic acids concentration, due to the highest content of oxalic and malic acids. This study is of great importance because it fills an existing void in relation to chemical characterization of these plants, more precisely its composition in sugars, organic acids, and tocopherols, that can be present in the consumed forms (mostly infusions).

## Figures and Tables

**Table 1:** Composition of *P. tridentatum*, *G. globosa*, and *C. citratus* in tocopherols, sugars, and organic acids (mean ± SD).

	*Pterospartum tridentatum *	*Gomphrena globosa *	*Cymbopogon citratus *
*α*-Tocopherol	7.21 ± 0.01^b^	0.38 ± 0.04^c^	56.05 ± 2.47^a^
*γ*-Tocopherol	5.81 ± 0.56^a^	3.02 ± 0.08^b^	4.52 ± 0.76^a^
*δ*-Tocopherol	0.50 ± 0.10^b^	5.20 ± 0.01^a^	nd
Total tocopherols (*μ*g/g dw)	13.10 ± 1.08^b^	8.60 ± 0.10^c^	60.57 ± 3.23^a^

Fructose	83.23 ± 7.71^a^	18.30 ± 1.27^b^	7.35 ± 1.06^c^
Glucose	26.70 ± 1.13^b^	15.65 ± 2.62^c^	29.75 ± 0.92^a^
Sucrose	23.75 ± 1.34^b^	nd	41.45 ± 0.21^a^
Total sugars (mg/g dw)	133.70 ± 7.50^a^	33.95 ± 3.89^c^	78.55 ± 2.19^b^

Oxalic acid	1.39 ± 0.02^b^	10.64 ± 0.04^a^	1.22 ± 0.15^b^
Malic acid	3.23 ± 0.90^b^	12.33 ± 0.55^a^	2.23 ± 0.10^b^
Ascorbic acid	nd	nd	0.24 ± 0.01
Shikimic acid	0.71 ± 0.01	nd	nd
Citric acid	5.99 ± 0.35^a^	2.40 ± 0.01^b^	nd
Succinic acid	nd	nd	10.29 ± 0.34
Fumaric acid	nd	0.28 ± 0.01^b^	0.49 ± 0.01^a^
Total organic acids (mg/g dw)	11.32 ± 1.26^c^	25.65 ± 0.51^a^	13.98 ± 0.47^b^

nd: not detected; dw: dry weight. In each row, different letters mean significant differences (*P* < 0.05).
